# Translation and psychometric properties of the tamil version of the smartphone addiction scale– short version (SAS-SV-T) among adolescents: measurement invariance across gender

**DOI:** 10.1186/s40359-025-03153-6

**Published:** 2025-08-06

**Authors:** Anbumalar C, Binu Sahayam D

**Affiliations:** School of Social Sciences and Languages, Vellore Institute of Technology, Kelambakkam - Vandalur Rd, Rajan Nagar, Chennai, Tamil Nadu 600127 India

**Keywords:** Problematic smartphone use, Youth mental health, Linguistic validation, Tamil speaking population, Scale adaptation, Adolescents, Internal consistency, Factor structure

## Abstract

**Background:**

Adolescent smartphone addiction is a growing public health concern that negatively impacts mental health and daily functioning. The Smartphone Addiction Scale -Short Version (SAS-SV), developed by Kwon and colleagues, is a widely validated measure used globally to assess this issue. However, a psychometrically sound Tamil version suitable for adolescents has been lacking. This study aimed to translate SAS-SV into Tamil, evaluate its psychometric properties, and assess measurement invariance across genders.

**Methods:**

The translation followed internationally accepted cross-cultural adaptation guidelines. Cronbach’s α and McDonald’s ω were used to measure reliability. The study employed Confirmatory Factor Analysis (CFA) to assess structural validity and Multigroup CFA (MGCFA) examined measurement invariance across gender.

**Results:**

Internal consistency for the SAS-SV-T is good (Cronbach’s α = 0.83 and McDonald’s ω is 0.83). The CFA results indicated standardized factor loadings of 0.49 to 0.61, supported by a unidimensional structure. The model fit was acceptable with χ²(df) = 58.7(34), *p* =.01; CFI = 0.98; TLI = 0.97; RMSEA = 0.04; SRMR = 0.03. The scale also showed full measurement invariance across gender.

**Conclusion:**

A valid and reliable measure of smartphone addiction in Tamil-speaking adolescents is the SAS-SV-T. Measurement invariance across gender supports its use in both research and clinical practice. This study fills a gap in adolescent health and addiction research by offering a culturally relevant instrument.

## Introduction

India is now one of the fastest-growing digital nations due to its extensive smartphone use and reasonably priced internet connectivity [1]. Studies show that Indian adolescents spend several hours daily on their smartphones for entertainment, gaming, and social networking, raising concerns about compulsive usage and potential addiction [2, 3].

The issue of whether excessive smartphone use can result in addiction has received considerable attention since the invention of smartphones [4]. Smartphones have become essential tools for many with multiple functions, such as messaging, calling, music, gaming, and internet access. Nonetheless, some individuals unknowingly become highly dependent on their devices. Kwon et al. (2013) broadened the definition of addiction to include behavioral addictions like gambling, internet addiction, gaming, and smartphone dependency [5]. Commonly used terms for smartphone addiction include smartphone addiction [5], mobile phone (smartphone) dependence [6], problematic smartphone use [7], excessive smartphone use [8], and nomophobia [9], where overuse of smartphones to the extent that it disturbs the users’ daily lives [10].

Adolescents growing dependence on smartphones in India has sparked worries about addiction and its impact on Mental Health. By 2025, over 900 million Indians will primarily use smartphones for the Internet, according to the Internet and Mobile Association of India (IAMAI) [11]. Research has shown varying prevalence rates of smartphone addiction among Indian adolescents. According to a study in Delhi, nearly one-third of teenagers showed symptoms of smartphone addiction [2], while another study in Gujarat reported an even higher prevalence, with 64.6% of school-going adolescents classified as people with an addiction [3].

Given the country’s fast digital change, more study is needed to ascertain smartphone addiction incidence, causes, and effects, particularly among teenagers [1]. The SAS-SV was developed as a methodical way to evaluate smartphone addiction and it has been extensively validated in different cultural contexts due to its briefness and effectiveness in measuring smartphone addiction severity. While the original SAS-SV was conceptualized as a unidimensional measure [5], some validation studies in other cultural contexts have reported alternative structures, including two factor [12, 13] or three factor model [14]. Despite these variations, the present study tested a unidimensional structure to maintain conceptual consistency with the original instrument and ensure comparability across global studies. However, the linguistic and cultural relevance of the psychometric tool to the target population is an important factor when using it.

Accurate and meaningful assessment requires translating the SAS-SV into regional languages. In Tamil Nadu, where a significant proportion of adolescents speak Tamil, a validated Tamil version of the SAS-SV is crucial. Directly using a tool developed in another language may lead to misinterpretations or culturally irrelevant findings [15]. Therefore, a linguistically and culturally adapted version clearly demonstrates that the scale appropriately captures the unique experiences and background of Tamil-speaking adolescents.

A crucial step in verifying the scale’s suitability for a diverse population is establishing measurement invariance across male and female respondents. Research on smartphone addiction has shown mixed results regarding gender differences. Some report that males are more susceptible to smartphone addiction [16], while others find no gender-based differences [17]. According to previous research, females are more likely than males to be addicted to smartphones [5, 18]. These discrepancies highlight the significance of establishing whether the SAS-SV similarly captures the concept of smartphone addiction across genders. Establishing measurement invariance is vital for ensuring that observed differences in scores are valid and not due to bias in the instrument [19]. Any comparisons based on gender could be deceptive if measurement invariance is not established.

### Significance of the study

The current study is significant as it addresses the adolescent mental health assessment within the Tamil-speaking population. The SAS-SV had no psychometrically validated Tamil version before this study, even though smartphone addiction is becoming more common among Indian adolescents. Given India’s linguistic and cultural diversity, depending only on English or other regional versions may lead to misinterpretation and reduced accuracy in assessment. The study provides a culturally sensitive, reliable, and efficient tool to screen smartphone addiction by translating and validating the SAS-SV into Tamil and providing its measurement invariance across gender. Having access to such a tool enables school counsellors, psychologists, and researchers to identify at-risk adolescents and provide interventions. Hence, the study aims to (a) systematically translate the SAS-SV into Tamil, (b) evaluate its psychometric properties, including reliability and factor structure, and (c) assess gender-based measurement invariance among adolescents in Chennai.

## Methods

### Participants and procedure

The study was conducted in Government and Government aided schools in Chennai between January and March 2025. The study protocol was approved by the Institutional Ethics Committee. A cross-sectional research design was employed for the study. Four hundred and forty adolescents between 12 and 15 years comprised the sample (Mean age 13.28 years, SD = 0.84), of whom 48.2% were male (*n* = 212) and 51.8% were female (*n* = 228). Parents, the participants, and the principal all gave their informed consent. Written informed consent was obtained from the school principal and parents, and assent was obtained from all participants. The researcher clearly explained the study purpose, voluntary participation, right to withdraw at any time, and confidentiality of their responses in age-appropriate language. Participation was entirely voluntary, and participants were assured that they could discontinue at any point without facing any consequences. All data were treated confidentially and used only for research purposes. The study was done in three phases based on its objectives. The first phase involved translating the instrument from English to Tamil by following the methodology suggested in the existing literature as well as the guidelines provided by Beaton et al. [15], In the second phase, the instrument was validated for the Tamil-speaking adolescents by evaluating the psychometric properties of the Tamil translated version of the tool. In the third phase, the Multigroup CFA was used to evaluate the measurement invariance across gender.

### Measures

The Smartphone Addiction Scale-Short Version (SAS-SV), originally developed by Kwon et al. [5], was used to assess smartphone addiction. The scale consists of 10 items; each rated on a 6-point Likert Scale ranging from 1 (strongly disagree) to 6 (strongly agree). The total score is obtained by summing up the scores of the items, with higher scores indicating a greater risk for smartphone addiction.

### Phase 1: translation procedure

Following the established protocols by Beaton et al. [15], the SAS-SV was cross-culturally adapted and translated into Tamil. The translation process began after obtaining email permission from the original author of the SAS-SV. The following were the steps involved in the translation process.

### Step 1:

Two independent bilingual translators proficient in both Tamil and English were aware of the cultural background of both languages and translated the SAS-SV English into Tamil. One translator had a background in psychology, and the other was a non-specialist, capturing layman and clinical perspectives. This process produced two Tamil translations.

### Step 2: synthesis of the translations

The two translators and an independent observer synthesized the results, producing one reconciled translation. Any ambiguity between the two translations was discussed item by item and resolved through discussion to ensure clarity, relevance, and linguistic simplicity.

### Step 3: back translation

The synthesized version was back translated into English by the two translators who were unfamiliar with the original SAS-SV. This procedure was used to identify and rectify any unclear terms in the translations and verify that they convey the same meaning as the original version.

### Step 4: expert committee review

An expert committee, including psychologists and linguistics professionals, compared and reviewed the translated versions. The committee then consolidated all questionnaire versions, resulting in a refined prefinal version of the Tamil SAS-SV for field testing.

### Step 5: tests of the prefinal version and cognitive debriefing

Forty Tamil-speaking adolescents aged 12 to 15 were asked to review the prefinal Tamil version to determine its cultural acceptability, clarity, and comprehension. After completing the scale in class, participants were interviewed using cognitive debriefing techniques. They were asked to provide feedback on each item’s clarity, their comprehension of the answers, and any terms that were unclear or unrelated to their culture. According to feedback, the items are culturally relevant and easily understood. There were no complaints, confirming the translation’s appropriateness and the participants completed answering the questions within one to three minutes.

### Phase 2: validation of the SAS-SV-T

The structural validity of SAS-SV-T was examined using Confirmatory Factor Analysis by Maximum Likelihood (ML) estimation in AMOS 23.0. Model fit was assessed using χ², CFI, TLI, RMSEA, and SRMR. Internal consistency was evaluated using Cronbach’s alpha (α) in SPSS 25.0 and McDonald’s omega (ω) in R version 4.5.0.

### Phase 3: measurement invariance of SAS-SV-T across gender

Measurement invariance across gender was examined using Multigroup Confirmatory Factor Analysis (MGCFA) in AMOS 23.0. The Tamil version of the SAS-SV demonstrated that both male and female adolescents measured the same construct comparably. Four nested models, configural, metric, scalar, and strict invariance, were employed stepwise to evaluate measurement invariance across gender [20].

Configural invariance examined whether the fixed and free factor loading patterns, representing the number of factors and the item-factor structure, were consistent between the male and female groups. This baseline model showed that both groups conceptualized the latent construct similarly and provided the basis for further comparisons [20].

Metric invariance (or weak factorial) was assessed by restricting the factor loadings to be equal for each gender. A meaningful comparison of the associations between the latent variable and external variables was made feasible by ensuring that each item in both groups contributed proportionately to the latent variable [20].

For scalar invariance (strong factorial invariance), item intercepts and factor loadings were constrained across the gender groups. Establishing scalar invariance was essential for making valid comparisons of latent means between male and female adolescents [20].

The most restrictive form, strict invariance, added equality constraints on the residual variances of the items across genders. This level of invariance confirmed that the measurement errors were comparable across groups, supporting full measurement equivalence [20].

### Statistical analysis

The Statistical analysis was carried out using SPSS 25.0, AMOS 23.0, and R version 4.5.0. Skewness and kurtosis values were utilized to evaluate normalcy, and descriptive statistics were employed to describe demographic information. The SAS-SV-T’s internal consistency was evaluated using Cronbach’s α and McDonald’s ω. In order to obtain a robust estimate of the 95% confidence interval (CI) for ω, a bootstrap procedure with 1000 replications was performed using the boot package in R.

Based on the accepted guidelines, 440 participants were chosen for performing CFA. In order to achieve model stability and generalizability, Hair et al. (2016) recommend that 10–20 participants be used for each item on a scale [21], whereas according to Kline [22], performing CFA requires at least 100–200 participants for basic CFA models but recommends larger sample (300 to 400 or more) for complex models. With ten SAS-SV-T items, the sample size is larger than recommended, offering enough power for multigroup studies like measurement invariance testing and model estimation. Furthermore, larger sample sizes increase the accuracy of parameter estimations and model fit indices when evaluating complex models [23]. The SAS-SV-T’s factor structure and structural validity were estimated using Maximum Likelihood (ML) in CFA. Although SAS-SV-T consists of ordinal items, ML was chosen based on multiple considerations. The six-point Likert response format meets the criteria for treating ordinal data as continuous in factor analysis [24], and all item distributions demonstrated normality with skewness and kurtosis values within acceptable changes (|skewness| < 3;|kurtosis| < 10) [22]. Additionally, the sample size of 440 provided adequate power for ML estimation. Consistency with prior SAS-SV validation studies that used ML estimation in different cultural contexts further supported this choice [13, 14, 25, 26]. The analysis evaluated whether the items supported the single latent factor characterizing smartphone addiction, supporting the unidimensional model previously established in other populations. The indices used to assess model fit included chi-square (χ²), degrees of freedom (df), Chi-square/df ratio (CMIN/DF), CFI, TLI, RMSEA, and SRMR. Hu and Bentler [27] suggest that a model is a good fit if χ² is non-significant, χ²/df is below 5, CFI and TLI exceed 0.95, and RMSEA and SRMR are below 0.08. Both the entire sample and gender-based subgroups were subjected to these standards.

The four hierarchically nested models (configural, metric, scalar, and strict) previously described were used to evaluate measurement invariance across gender. Males, females, and the entire sample were first subjected to separate single-group CFAs. Changes in fit indices were used to compare models. Using ΔCFI and ΔRMSEA, changes in model fit were assessed; values < 0.01 and ≤ 0.015, respectively, were deemed acceptable [19]. While Rutkowski and Svetina [28] proposed a more lenient threshold (e.g., ΔRMSEA < 0.050) for more complex multi-group models, the present study adheres to the strict criteria for a two-group comparison. Chi-Square difference was reported for completeness, though not used as a primary criterion due to its sensitivity to sample size [19, 29].

## Results

### Descriptive statistics

Table 1 summarizes descriptive statistics for the participants. The average age of the 440 participants was 13.28 years (SD = 0.84). The gender distribution of the participants was 48.2% male and 51.8% female. The majority of the participants were in Grade 8 (46.1%), followed by Grade 9 (30.5%) and Grade 7 (23.4%). All the participants resided in urban areas and used smartphones daily. The average daily screen time was 2.4 h (SD = 2.61).

**Table 1:** Participants characteristics

Variable		*N*	%	Mean	SD
Age (in years)		440		13.28	0.84
Gender	Male	212	48.2		
	Female	228	51.8		
Education	7th	103	23.4		
	8th	203	46.1		
	9th	134	30.5		
Residential Status	Rural				
	Urban	440			
Average Daily Screen Time (hours)		440		2.4	2.61

### Item descriptive statistics of SAS-SV-T

Table 2 shows the SAS-SV-T item-level descriptive statistics. For the overall sample, the standard deviations ranged between 0.981 and 1.086, while the mean item scores ranged from 2.22 to 4.87. The mean item scores for males and females ranged from 2.10 to 4.86 and 2.32 to 4.87, respectively. The absolute values for skewness and kurtosis of the items were below 2, indicating approximate normality [22].

**Table 2:** Item level descriptive statistics for SAS-SV-T by gender [Overall, Male, Female]

Item	Mean	Standard Deviation	Skewness	Kurtosis
1	3.31 (3.31,3.30)	1.086 (1.034,1.134)	0.075 (-0.082,0.187)	− 0.237 (-0.205, − 0.275)
2	2.48 (2.48,2.48)	1.019 (0.976,1.060)	0.268 (0.129,0.361)	− 0.574 (-0.714, − 0.497)
3	2.22 (2.10,2.32)	1.002 (0.968,1.024)	0.642 (0.613,0.656)	0.091 (0.272,0.315)
4	3.83 (3.81,3.86)	1.020 (1.099,0.942)	− 0.180 (-0.236, -0.67)	0.053 (-0.004, − 0.038)
5	4.87 (4.86,4.87)	0.981 (0.916,1.040)	− 0.606 (-0.360, − 0.763)	0.068 (0.734,0.473)
6	4.38 (4.41,4.35)	1.067 (1.038,1.094)	− 0.441 (-0.349, − 0.509)	0.012 (0.004,0.006)
7	4.51 (4.50,4.51)	1.019 (1.028,1.013)	− 0.317 (-0.369, − 0.267)	− 0.329(-0.180, − 0.458)
8	3.25 (3.12,3.37)	1.063 (0.979,1.125)	0.024 (0.147,0.049)	− 0.162 (0.375, − 0.184)
9	2.78 (2.70,2.85)	1.035 (1.032,1.034)	0.159 (0.162,0.158)	− 0.436 (-0.581, − 0.295)
10	4.20 (4.15,4.25)	1.024 (1.013,1.034)	− 0.166 (0.104, − 0.228)	− 0.180 (-0.141,0.170)

### Confirmatory factor analysis

#### CFA for overall sample

**Fig. 1:**
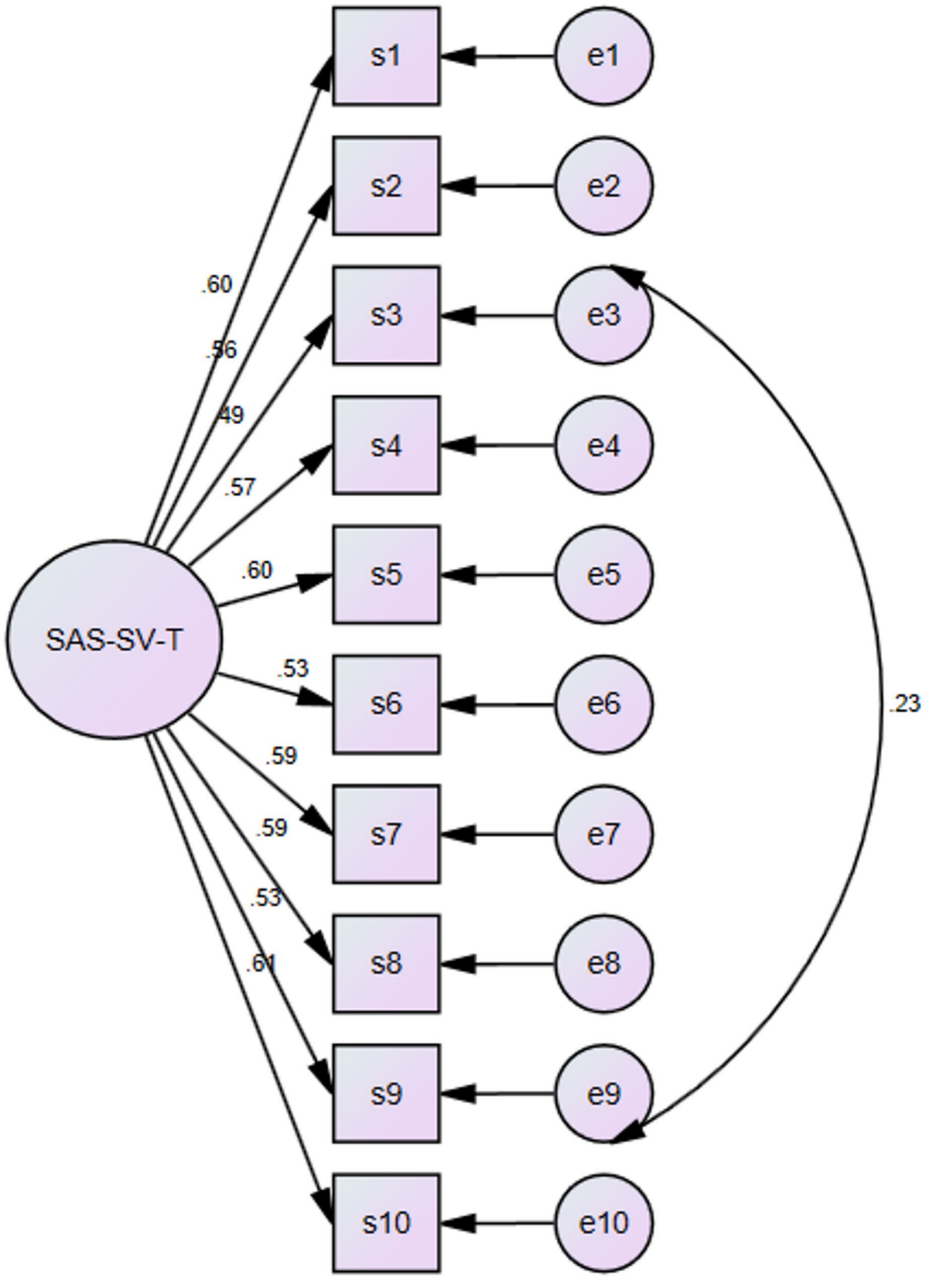
The path diagram of the SAS-SV-T

CFA was performed in a single group to evaluate the SAS-SV-T structural validity using ML. The initial one factor model yielded adequate fit indices: χ^2^(35) = 78.59, χ^2^/df = 2.25, CFI = 0.96, TLI = 0.94, RMSEA = 0.05, SRMR = 0.03. Table 3 illustrates that the CFI exceeded the conventional cut-off of 0.95 [27], and the TLI was slightly below the recommended threshold of 0.95, suggesting the model fit could be improved. Inspecting the modification indices (MIs) showed that the error terms of Item 3 and Item 9 had a statistically and theoretically meaningful covariance (MI = 18.88). Although the contents of these items differ superficially- addressing physical symptoms and behavioral overuse, respectively both reflect excessive and prolonged smartphone usage, which can manifest both physically and behaviorally in adolescents. Prior studies have also reported that somatic symptoms such as musculoskeletal pain can be secondary effects of extended, compulsive smartphone engagement [30]. Therefore, allowing the covariance between their error terms was deemed theoretically defensible. In order to evaluate the updated CFA model, a covariance path between Item 3 and 9’s error terms was added. Improved fit was demonstrated by this adjusted model: χ² (34) = 58.7, *p* =.01, χ²/df = 1.73, CFI = 0.98, TLI = 0.97, RMSEA = 0.04, SRMR = 0.03. The unidimensional structure of the scale was supported by all standardized factor loadings, which varied from 0.49 to 0.61. The final CFA model is illustrated in Fig. 1.

**Table 3:** CFA model fit indices for SAS-SV– T

Model/Group	χ^2^	*p*	df	χ^2/^df	CFI	TLI	RMSEA	SRMR
Original SAS-SV	78.59	< 0.001	35	2.25	0.96	0.94	0.05	0.03
Modified SAS-SV	58.70	0.01	34	1.73	0.98	0.97	0.04	0.03
All Participants (*n* = 440)	58.70	0.01	34	1.73	0.98	0.97	0.04	0.03
Male (*n* = 212)	46.90	0.09	35	1.34	0.98	0.97	0.04	0.03
Female (*n* = 228)	46.95	0.07	34	1.38	0.98	0.97	0.04	0.03

χ^2^ = Chi-Square, p = p value, χ^2/^df = Chi-square likelihood ratio statistics, CFI = comparative fit index, TLI = Tucker-Lewis Index, RMSEA = root mean square error of approximation, SRMR = standardized root mean square residual

### CFA for male subgroup

The model demonstrated a good fit for the male subgroup (*n* = 212), with the following results: χ² (35) = 46.90, *p* =.09, χ²/df = 1.34, CFI = 0.98, TLI = 0.97, RMSEA = 0.04, and SRMR = 0.03. Notably, without the requirement for any additional error covariances, indicating that the unidimensional structure was well-supported without model modification. All the standardized factor loadings were consistent with the sample, ranging from 0.50 to 0.62.

### CFA for female subgroup

In the analysis of the female subgroup (*n* = 228), the initial CFA model produced a χ² (35) = 74.16, *p* =.00, χ²/df = 2.12, CFI = 0.93, TLI = 0.91, RMSEA = 0.07, and SRMR = 0.05. After reviewing the modification indices and considering theoretical relevance, a covariance was added between the error terms of Items 3 and 9. The updated model yielded satisfactory fit indices: χ² (34) = 46.95, *p* =.0.07, χ²/df = 1.38, CFI = 0.98, TLI = 0.97, RMSEA = 0.04, SRMR = 0.03. Standardized factor loadings for the female participants indicated significant item contributions from the items, which ranged from 0.45 to.64.

Allowing correlated error terms CFA is acceptable when supported by theoretical justification and statistical evidence, as it helps capture unexplained shared variance not accounted for by the latent construct [20, 31].

### Internal consistency of SAS-SV-T

The overall sample’s Cronbach’s α and McDonald’s ω for the SAS-SV-T were both 0.83, indicating good internal consistency. The reliability of the scale was further supported by a bootstrap study with 1000 replications, which produced a 95% CI for ω of 0.80 to 0.85. Subgroup analyses further supported this, with α = 0.82 and ω = 0.82 for males, and α = 0.84 and ω = 0.84 for females, demonstrating consistent reliability across genders. All items had corrected item-total correlations > 0.50 except items 2, 3, and 6, which were slightly low. However, if any item was deleted, all alpha values remained > 0.80, supporting the reliability across genders.

### Measurement invariance across gender

Table 4 displays the results.

The measurement invariance tests were carried out using AMOS to determine whether the smartphone addiction scale performs similarly for each gender. The results showed that the Configural Invariance Model (M1), the baseline model without equality restrictions, matched the data effectively (χ^2^ = 121.06, df = 70, χ^2^ /df = 1.73, RMSEA = 0.04, CFI = 0.95, SRMR = 0.03). Hence, Configural invariance was demonstrated across groups.

Metric invariance (M2) was examined by applying equality constraints to the factor loadings across genders. The model also showed a good fit (χ^2^ = 130.95, df = 80, χ^2^ /df = 1.64, RMSEA = 0.04, CFI = 0.95, SRMR = 0.03) with a negligible change in fit compared to the configural model (Δ χ^2^ = 9.89, Δdf = 10, ΔCFI = 0.00, ΔRMSEA = 0.00). The SAS-SV-T metrics gender invariance was supported.

With the addition of intercept constraints, the scalar invariance model maintained an adequate fit (χ^2^ = 147.50, df = 90, χ^2^/df = 1.64, RMSEA = 0.04, CFI = 0.94, SRMR = 0.03), with minimal change from the metric model Δχ^2^ = 16.55, Δdf = 10, ΔCFI=-0.01, ΔRMSEA = 0.00. Finally, the strict invariance model (M4) produced identical fit statistics as the scalar model (χ^2^ = 159.04, df = 100, χ^2^ /df = 1.59, RMSEA = 0.04, CFI = 0.94, SRMR = 0.03) and included equal residual item variances across genders, supporting the establishment of full measurement invariance (Δ χ^2^ = 11.54, Δ df = 10, ΔCFI = 0.00, ΔRMSEA = 0.00). As a result, it was confirmed that SAS-SV-T exhibited strict invariance across genders.

**Table 4:** Measurement invariance tests across gender

	Model Fit Indices						Model Difference					
Model	χ^2^	df	χ^2^ / df	RMSEA	CFI	SRMR	ΔM	Δ χ^2^	Δ df	ΔRMSEA	ΔCFI	ΔSRMR
M1	121.06	70	1.73	0.04	0.95	0.03	-	-	-	-	-	-
M2	130.95	80	1.64	0.04	0.95	0.03	M2 vs. M1	9.89	10	0.00	0.00	0.00
M3	147.50	90	1.64	0.04	0.94	0.03	M3vs.M2	16.55	10	0.00	-0.01	0.00
M4	159.04	100	1.59	0.04	0.94	0.03	M4vs.M3	11.54	10	0.00	0.00	0.00

### Group differences in smartphone addiction scores

An independent t-test was used to compare the two groups’ total smartphone addiction scores. The findings showed no significant difference between the two groups, t (438) = -1.20, *p* =.23 (2-tailed), indicating that smartphone use was comparable across genders.

## Discussion

The primary objective of the study was to translate and validate the SAS-SV-T and evaluate its measuring invariance between gender among adolescents in Chennai, India. This study fills a significant gap by offering a psychometrically sound and culturally adapted assessment tool for early identification and intervention activities, given the fast digitalization and increasing reliance of Indian teenagers on smartphones [2]. The results support the unidimensional structure, internal consistency, and gender invariance of the SAS-SV-T, establishing it as a reliable indicator for populations of Tamil speakers.

The single-group CFA indicated a good fit for the proposed one-factor model. A strong latent construct of smartphone addiction was reflected in all standardized factor loadings, which were statistically significant and above the traditional cutoff of 0.50 [21]. The model fit was marginally improved by including a covariance between Items 3 and 9. This aligns with previous research that found item-pair correlations [18, 32, 33]. These findings are consistent with validation results from other cultural contexts, Iran [26] and China [18, 34], all of which used CFA to confirm the SAS-SV’s unidimensional structure.

In the Tamil version, the overall sample’s Cronbach’s α and McDonald’s ω, both measures of internal consistency, were 0.83, indicating good reliability. Additionally, the 95% CI for ω, which is 0.80 to 0.85, provides more empirical support for the scale’s reliability. Subgroup analyses further supported the scale’s reliability, with α = 0.82 and ω = 0.82 for males and α = 0.84 and ω = 0.84 for females. These results are in good agreement with those found in international validation studies conducted in Spain (α = 0.88) [32], Turkey (α = 0.88) [35], and Brazil (α = 0.81) [36], indicating that the SAS-SV maintains its reliability in a variety of linguistic and cultural contexts.

One of the study’s main strengths was the measurement invariance testing across gender. We used multi-group CFA to analyse configural, metric, scalar, and strict invariance. The model satisfied every criterion (ΔCFI < 0.01, ΔRMSEA < 0.015), demonstrating that the SAS-SV-T performs similarly for Tamil Nadu’s male and female adolescents. These findings align with Yue et al. [34], who confirmed the SAS-SV’s gender invariance in Chinese university students. Servidio et al. [37] reported that the Italian version of the SAS-SV had configural and metric invariance across gender and partial scalar invariance, further supporting the scale’s cross-cultural applicability. Therefore, our full invariance results support the global psychometric foundation of the SAS-SV by providing a convincing example of gender equivalency in an Indian adolescent population. Thus, by offering an example of gender equivalency in an Indian adolescent population, our full invariance results strengthen the SAS-SV’s cross-cultural generalizability.

There were no significant gender differences in smartphone addiction scores among Tamil adolescents in the independent t-test analysis. The study found no statistically significant gender differences in mean SAS-SV-T scores. This finding aligns with recent evidence of gender balance in adolescents’ smartphone use patterns. For instance, Wu and Chou [38] and Guimarães et al. [39] reported no significant correlation between gender and smartphone addiction among adolescents. However, this result contradicts previous research that found that females were more prone to be addicted to smartphones. According to Sajidah et al. [40] and Sánchez-Martínez and Otero [41], smartphone addiction was more common among female adolescents. However, other studies suggest that boys are more prone than girls to become addicted to smartphones. Soleymani et al. [42] reported that male undergraduates exhibited higher levels of smartphone addiction than females. Although there may be gender differences in content preferences (e.g., social media vs. gaming) [43], this study indicates that addictive symptoms are similar for both genders.

The large and diverse sample (*N* = 440), which includes both genders and is drawn from several urban Chennai schools, further supports the SAS-SV-T’s robustness. Smartphone ownership and usage have skyrocketed among Indian adolescents [44], and studies have connected excessive use to mental health issues [45], academic problems, and sleep disturbances [46, 47], further supporting the scale’s applicability in this demographic.

With a slight adjustment—introducing covariance between Items 3 and 9—the Tamil version of the SAS-SV showed a strong unidimensional structure and excellent model fit, resulting in optimal fit indices. This indicates that the translated materials have conceptual clarity and cultural resonance among adolescents who speak Tamil. The thorough translation methodology that ensures contextual equivalency and linguistic appropriateness through forward and backward translation processes and pre-testing with the target age group may be responsible for this clarity.​.

### Limitations

The results may not be generalized to rural and semi-urban populations as it was limited to adolescents aged 12–15 from urban government and government-aided schools in Chennai. Only self-reported information was used, which might be biased due to social desirability or inaccurate recall. The study excluded non-binary and gender-diverse adolescents by including only male and female participants. Cross-sectional data limits conclusions about causality or changes in addiction over time. Furthermore, the study did not compare SAS-SV-T scores with theoretically linked constructs like sleep quality, anxiety, or screen time behavior to examine convergent, concurrent or criterion validity. Despite demonstrating scale’s strong internal consistency, acceptable factor structure, and gender invariance, the absence of external validity evidence limits the full assessment of construct validity in this specific cultural setting.

### Future directions

Future studies should adopt more inclusive and different methodologies to improve the scope of the SAS-SV-T scale. It will be helpful to include rural, tribal, and economically disadvantaged adolescents will be useful. Longitudinal studies are needed to evaluate SAS-SV-T’s temporal stability and predictive validity over time. Additionally, correlating SAS-SV-T scores with related constructs such as anxiety, sleep quality, or screen-time behavior will help establish convergent, concurrent, and criterion validity. Using qualitative techniques will offer a deeper understanding of the lived experiences that underlie the symptoms of addiction.

## Conclusion

The Tamil Smartphone Addiction Scale– Short Version (SAS-SV-T) exhibits good psychometric properties and gender-neutral measurement. It is a valuable tool for researchers and clinicians looking to evaluate and treat smartphone addiction in Tamil-speaking adolescents because of its unidimensional structure, strong internal consistency, and confirmed gender invariance. Validated instruments like the SAS-SV-T are crucial for early detection, comparative study, and the creation of culturally sensitive interventions as smartphone usage in India keeps rising.

## Data Availability

The datasets used and/or analysed during the current study are available from the corresponding author on reasonable request.

## Abbreviations

SAS-SV-T: Smartphone Addiction Scale– Short Version-Tamil
SAS-SV: Smartphone Addiction Scale -Short Version
CFA: Confirmatory Factor Analysis
MGCFA: Multigroup Confirmatory Factor Analysis
IAMAI: Internet and Mobile Association of India
Df: Degrees of freedom
CFI: Comparative fit index
TLI: Tucker-Lewis Index
RMSEA: Root mean square error of approximation
SRMR: Standardized root mean square residual
SD: Standard Deviation
CMIN/DF: Chi-square/df ratio
MI: Modification Indices
ML: Maximum Likelihood
CI: Confidence Interval
